# Effect of Different Pretreatment Methods on Birch Outer Bark: New Biorefinery Routes

**DOI:** 10.3390/molecules21040427

**Published:** 2016-03-30

**Authors:** Anthi Karnaouri, Ulrika Rova, Paul Christakopoulos

**Affiliations:** Biochemical Process Engineering, Division of Chemical Engineering, Department of Civil, Environmental and Natural Resources Engineering, Luleå University of Technology, Luleå 971 87, Sweden; anthi.karnaouri@ltu.se (A.K.); Ulrika.Rova@ltu.se (U.R.)

**Keywords:** *Betula pendula*, outer bark, steam explosion pretreatment, organosolv pretreatment, suberin, chemical composition

## Abstract

A comparative study among different pretreatment methods used for the fractionation of the birch outer bark components, including steam explosion, hydrothermal and organosolv treatments based on the use of ethanol/water media, is reported. The residual solid fractions have been characterized by ATR-FTIR, ^13^C-solid-state NMR and morphological alterations after pretreatment were detected by scanning electron microscopy. The general chemical composition of the untreated and treated bark including determination of extractives, suberin, lignin and monosaccharides was also studied. Composition of the residual solid fraction and relative proportions of different components, as a function of the processing conditions, could be established. Organosolv treatment produces a suberin-rich solid fraction, while during hydrothermal and steam explosion treatment cleavage of polysaccharide bonds occurs. This work will provide a deeper fundamental knowledge of the bark chemical composition, thus increasing the utilization efficiency of birch outer bark and may create possibilities to up-scale the fractionation processes.

## 1. Introduction

Efficient bioconversion of lignocellulosic materials to ethanol and value-added biochemicals represents a challenging proposition from both energy and environmental viewpoints. Apart from polysaccharides, multiple biomass feedstocks, including cork and outer bark residues, are rich in natural waxes, such as triterpenes, and insoluble polyesters (suberin) that serve as structural components of the outer barriers of plants. In order to improve the utilization of these feedstocks, an in depth delineation of the extraction and biodegradation of these components is needed. Moreover, degradation products, some of which are found almost exclusively in the suberized plant cell walls, may be of considerable value as sources of oleochemicals [1,2,3].

Silver birch (*Betula pendula*) is widely distributed in the northern hemisphere, particularly in boreal climates, and is of great commercial significance as it constitutes the dominant hardwood tree species used for pulp production in Northern European countries. The total production of market pulp in Sweden amounts to approximately 3.8 million tons annually, according to The Swedish Forest Industries Fact and Figures, leading to the production of considerable amounts of birch bark as a residual product from log debarking, usually burned for energy production. According to the biorefinery concept, using biomass directly as solid fuel is a waste of valuable material. Instead, the natural and renewable resources should be used to prepare value-added products such as chemicals and materials [4,5]. The bark of birch tree has been the subject of intensive research due to its high content of compounds with wide beneficial chemistry and bioactivity, such as pentacyclic lupin-type triterpenes and suberinic polyesters [6,7].

Bark, the external layer that surrounds the stem, branches and roots, can be roughly divided into inner bark, comprised of living cells that still maintain the function for transportation of liquids and nutrients, and the dead tissues of the outer bark or rhytidome [8]. The outer bark consists mainly of a periderm of cork layers forming an apoplastic barrier that controls the flow of water, gases and ions, thus protecting the plant against pathogens. The walls of these cork cells are impregnated with suberin, a lipid-derived insoluble polyester. Suberin consists of a lignin-like polyaromatic domain formed by hydroxycinnamic acids and a cutin-like polyaliphatic domain composed of hydroxy or epoxy fatty acids joined by ester linkages [9], but its complete structure remains unknown. Bark also contains high content of extractable constituents (extractives) including organic solvent and water soluble fractions (triterpenes and phenolic compounds) [10], so its chemistry can be practically subdivided into the chemistry of the extractives and the chemistry of suberin. Both of these classes of molecules can be used as starting materials in the synthesis and production of different poly- and oligomeric value-added products, such as polyols, and polyurethanes [11,12]. Besides that, bark extractives find numerous applications in pharmaceutical and cosmetic industry as antioxidants, due to their bioactive characteristics, such as anti-virus, anti-inflammatory, anticancer and other properties [13,14].

An integral key step in all the biotechnological technologies employed for the exploitation and valorization of lignocellulosic biomass, is the application of an initial pretreatment that will convert raw materials to a form amenable to enzymatic degradation [15]. Chemical or thermochemical pretreatments facilitate the disruption of the secondary cell walls structure and reduce biomass recalcitrance in various ways, including reduction in crystallinity and degree of polymerization of cellulose, lignin and hemicellulose modification and/or degradation and increase of pore volume [16,17]. It also allows for the partial or more thorough fractionation of polysaccharidic and phenolic cell wall components that can be more easily processed downstream. Hydrothermal (HT) and steam explosion (SE) pretreatments have been shown to lead to rupture of the biomass fibers’ rigid structure [18,19]. During steam explosion, biomass is treated with hot steam (180–240 °C) under pressure (1–3.5 MPa) followed by an explosive decompression, whereas during hydrothermal pretreatment, water is present as a liquid. Biomass modifications after steam explosion are related to hydrolysis and deacetylation of hemicellulose, partial removal of lignin, defibrillation of cellulose bundles and disruption of fibers [17]. Organosolv (OS) fractionation, in which organic solvents or their aqueous solutions are used as the pretreatment medium, yields three separate fractions including dry lignin, an aqueous hemicellulose stream, and a relatively pure cellulose fraction [16]. This method offers the advantage of hydrolysis and dissolution of aromatic structures, which can occur readily than with water-rich counterparts, because of the penetration of the organic liquor into the cell wall [18]. Moreover, organic solvents can be easily recovered through distillation and recycled. It occurs usually at high temperatures (140–180 °C) in the presence or absence of a catalyst. The use of alkaline catalyst along with organic solvents is often investigated, as alkaline catalysts have been shown to have significant effect on disruption of ester bonds between lignin and hemicellulose, thus achieving delignification, as well as removal of acetyl/uronic acids from hemicellulose [19].

Towards the valorization of bark as a residue from paper pulp mills in Sweden, in this study, *B. pendula* outer bark samples were subjected to different pretreatment methods and the effects of different process variables were studied. With the objective to analyze the potential of each pretreatment method for selective component enrichment within a bark valorization chain for energy, composite materials and chemicals, the major features of the residual solid fractions in relation to their microscopic structure and chemical properties were also evaluated

## 2. Results

### 2.1. Pretreatment and Bark Solubilization

Table 1 shows the results of different processing conditions, hydrothermal, steam explosion and organosolv treatment of birch outer bark in terms of severity factor and the percentage of extracted/solubilized material. The highest yield of dissolved substances in uncatalyzed treatments was obtained with organososlv treatment, with a ratio of 80:20 of ethanol–water mixture. The addition of 0.1 M sodium hydroxide resulted in a prominent increase of bark solubilization reaching a yield of 61.25% of the initial dry mass.

### 2.2. Chemical Characterization of the Residual Bark

#### 2.2.1. Overall Chemical Composition

The chemical composition of the residual solids and the liquid fractions are shown in Table 2 and Table 3. Total extractives of untreated material reach 39.4%, corresponding mainly to waxes and non-polar compounds, extracted with dichloromethane. Polar compounds correspond to 35% of total extractives. After pretreatment, there is a decrease in total amount of extractives, mainly attributed to the ethanol and water extractives, while non-polar compounds percentage is not affected. In organosolv samples, the amount of total extractives appears significantly lower, along with the increase of ethanol concentration in liquid mixture of pretreatment.

The monomeric composition of polysaccharides, which corresponds only to 4.45% of untreated birch outer bark, is given in Table 2 in relation to the proportion of total dry weight of the sample. It shows a predominance of glucose (50% of total neutral monosaccharides) and a significant content of arabinose (27%). The content of xylose is also comparatively high (15%). Detectable amounts of sugars originating from the hemicellulose were observed in the solid fraction after steam explosion pretreatment and at low concentration of ethanol, whereas the increase of the organic solvent concentration results largely to more extensive dissolution of the hemicelluloses. Suberin constitutes the 44.06% of the untreated outer bark. It is decreased after all types of pretreatment, with the highest degree detected upon employment of alkaline catalyst along with the organic solvent in the liquid mixture. Lignin constitutes approximately 9% of the initial material and its content increases remarkably after pretreatment. Both Klason and acid-soluble lignin may be overestimated for all solid residues due to possible interference from other non-lignin components [20]. The data resulted in a summative mass closure of 84%–98%, likely due to some components not accounted for in the analysis, for example pectin that would be detected as uronic acid [21] or hemicellulose acetyl groups which were also not quantified. The bark samples after organosolv fractionation gave lower mass closures, possibly due to residual extractives/condensed materials which were still present even after the subsequent extraction steps.

The composition of liquid fractions is reported in Table 3. Untreated outer bark contains a low amount of carbohydrates and thus their concentration in the liquid fraction (expressed in monomeric form) is negligible. Steam explosion stimulates the release of hemicellulosic sugars (xylose and arabinose), while they are detected in higher contents with increasing proportions of ethanol in organosolv treatments. The amount of phenolic compounds follows a similar pattern. Following the low initial sugar content of outer bark, degradation products generated during the pretreatments were also detected in traces, apart from acetic acid concentration that was higher in HT, OS/SE and OS/NaOH samples.

#### 2.2.2. ATR-FTIR Studies

**Figure 1:**
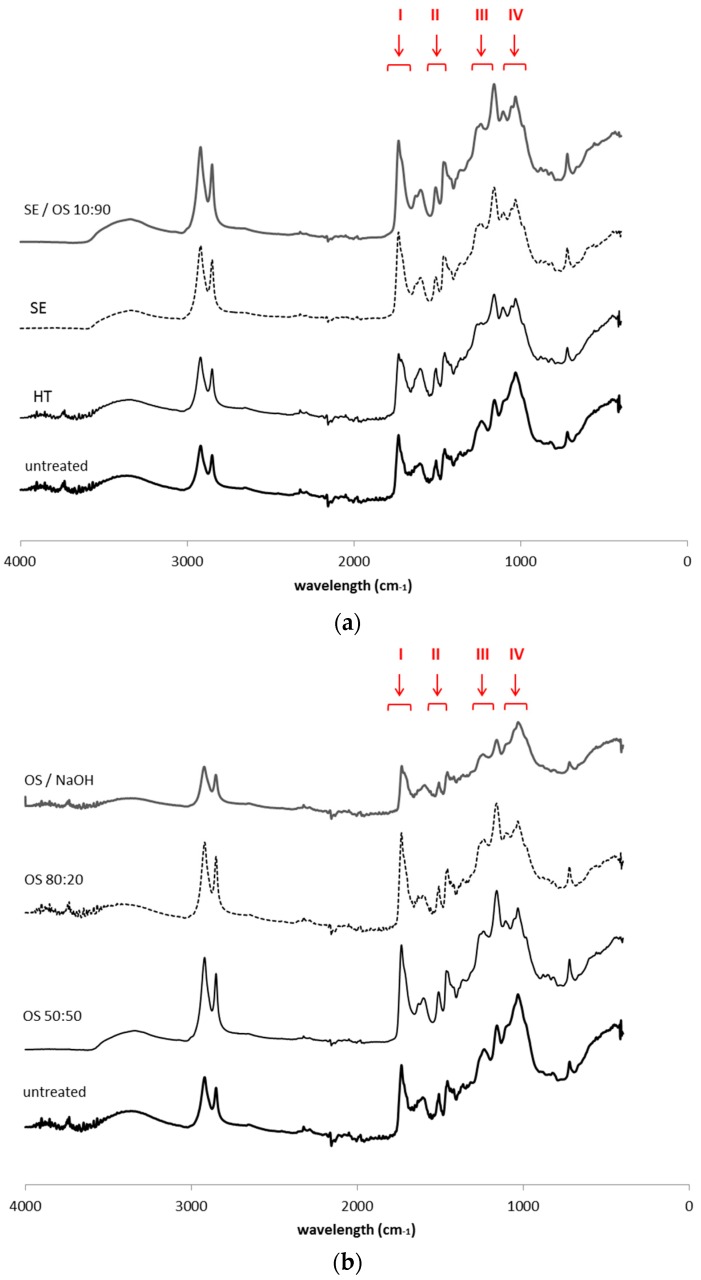
ATR-FTIR spectra of extractive—free untreated and pretreated solid fractions from (**a**) hydrothermal and steam explosion pretreatments and (**b**) treatments with organic solvents. The following regions marked correspond to: (**I**) region 1740–1715 cm^−1^ that accounts for the ester groups (C=O stretch); (**II**) “lignin triplet” region, including the three characteristic bands at 1600–1400 cm^−1^, derived from C=C vibrations of the aromatic ring, (**III**) region 1265–1161 cm^−1^ for symmetric and asymmetric C–O–C stretching and (**IV**) region 1092–1034 cm^−1^ that accounts for C–H, C–O and –OH deformations.

The spectrum of untreated bark samples is characterized by a very broad O–H stretch band at 3415 cm^−1^, attributed to carboxylic acids and alcohol groups, and two major bands at 2935 and 2850 cm^−1^ mainly attributed to the aliphatic moieties of suberin, corresponding to asymmetric and symmetric C–H stretching vibrations, respectively (Figure 1) [22,23,24,25]. The intense C=O stretching band at 1740 cm^−1^ accounts for the ester groups in suberin. This band shows a small shoulder at 1715 cm^−1^ that is usually associated with the C=O group of free carboxylic acids [26]. The 1636–1603 cm^−1^ region corresponds most probably to C=C stretch from the conjugated carbonyl groups of aromatic components that are part of the suberin and lignin structure, not excluding the possibility to result from the associated water molecules. The 1513 cm^−1^ band accounts for aromatic C=C stretching vibrations mainly from lignin components. The bands at 1468 cm^−1^ and 1364 cm^−1^ reflect C–H asymmetric and symmetric deformations of aliphatic regions, respectively. Bands at 1265, 1161 cm^−1^, corresponding to symmetric and asymmetric C–O–C stretching and at 722 cm^−1^ corresponding to C–H bend, all associated with vinyl groups, were also observed and attributed to suberin [27]. Polysaccharides contribute to absorption of the region 1092 and 1034 cm^−1^ with C–H, C–O and C–OH deformations. Bands at 855 and 819 cm^−1^ are associated with C-H deformation and ring vibration of lignin components.

#### 2.2.3. NMR Studies

The analysis of the ^13^C CP/MAS NMR spectra was conducted on the basis of previous work reported [22,24,28,29,30,31] and the main tentative assignments of the observed bands are presented in Table 4. The most intense aliphatic bands, at 30 and 33 ppm, correspond to aliphatic groups in suberin structure, with the 33 ppm carbons accounting for methylenes near the –CH_2_O– linkages between aliphatic suberin and the cell-wall matrix. The signal at 56 ppm arises mainly from lignin and suberin –OCH_3_ groups and epoxy rings but also methoxy groups from polysaccharide part, especially hemicellulose, can also contribute. The bands at 72–74 ppm arise from overlapping signals of carbohydrates and lignin aliphatic carbons. 104 ppm carbons have been suggested to correspond to polysaccharides, while in the 144–145 ppm region, signals arise from aromatic ring carbons from lignin and/or suberin. The band at 172 ppm corresponds to acetyl groups of suberin and hemicellulose as well as from carboxyls of lignin and suberin aromatic structures.

**Figure 2:**
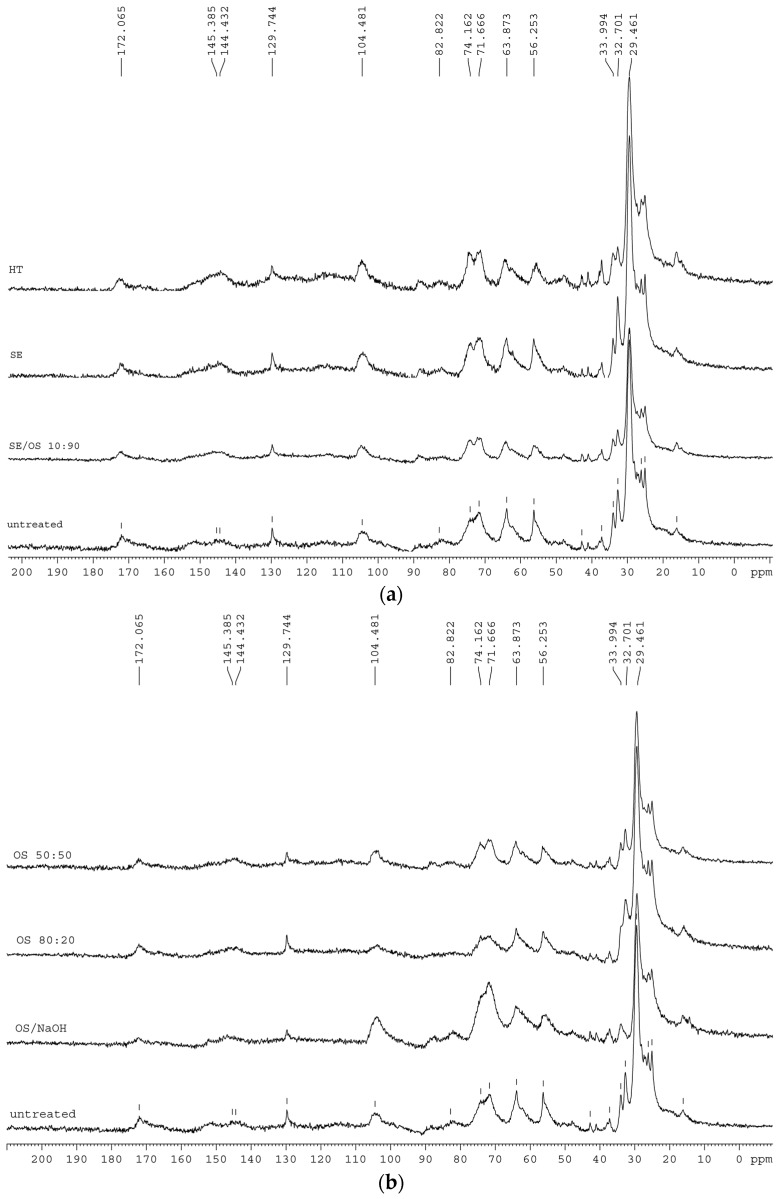
^13^C-CP/MAS NMR spectra extractive—free untreated and pretreated solid fractions from (**a**) hydrothermal and steam explosion pretreatments and (**b**) treatments with organic solvents.

The NMR spectra of pretreated residual solids (Figure 2) show that both main aliphatic bands generally decrease. In SE and OS/EtOH 10:90 and OS/EtOH 50:50, the two bands are affected in a similar way, but when higher ethanol concentration is used, the decrease at 30 ppm is slightly more significant than that at 33 ppm. When NaOH is added, the opposite effect is observed, thus confirming the preferential removal of methylenes at 33 ppm compared to those at 30 ppm. Reasonance from 33 ppm carbons disappears completely, while the band at 30 ppm has been decreased significantly. Strong intensity of 72–74 ppm bands is obvious in sample treated with alkaline catalyst. Reasonances from epoxy rings and methoxy groups are less intense in SE sample, but are not affected in the other spectra. Band at 104 ppm appear slightly increased in HT sample, but reduce when organic solvent is used in the liquid mixture; interestingly, a remarkable increase of this band is observed in spectrum of OS/NaOH sample. The band at 130 ppm appears in lower absorbance in all OS samples, especially when alkaline catalyst is used. Resonances from ester and carboxylic acid groups in 172 ppm remain unaltered in all samples, apart from those after HT and OS/NaOH treatment, indicating that part of these components is affected by pretreatment.

### 2.3. Morphological Characterization of the Residual Bark

**Figure 3:**
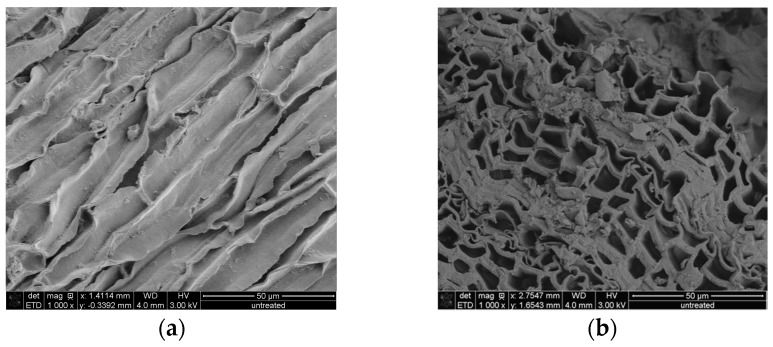
SEM images of (**a**) radial and (**b**) cross section of untreated birch outer bark. Magnification 1000×.

**Figure 4:**
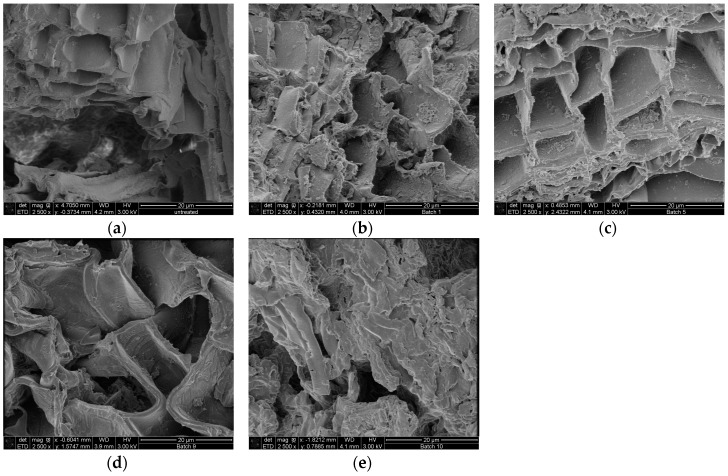
SEM images of cross section birch outer bark showing the effect of each type of pretreatment on the structural configuration of cell layers. (**a**) untreated material; (**b**) hydrothermal pretreatment (HT); (**c**) steam explosion (SE); (**d**) organosolv pretreatment without catalyst and (**e**) organosolv pretreatment with alkaline catalyst. Magnification 2500×.

**Figure 5:**
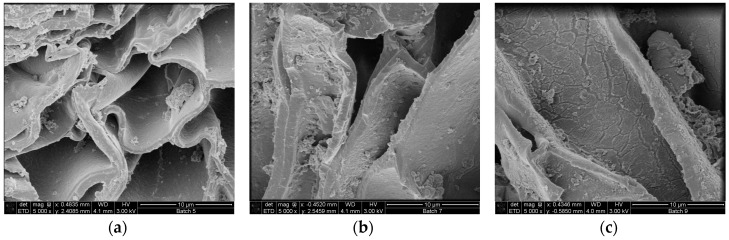
SEM images of birch outer bark showing cell wall deconstruction after organosolv treatment with increasing EtOH/water ratio in the liquid mixture (**a**) 10:90; (**b**) 50:50 and (**c**) 80:20. Magnification 5000×.

Scanning electrons microscopical observations of birch outer bark confirmed the existence of numerous cork (phellem) layers with an elongated polygonal shape (Figure 3a,b). After pretreatment, cell walls were ruptured to different extents. In case of hydrothermal and steam explosion treatment, cell walls are covered with residues (Figure 4b,c). Fragments of broken cell-walls also appear at some extent in all pretreatments of bark, illustrating the typical changes in structure of pretreated biomass. Surface residues markedly decrease as samples undergo organosolv treatments (Figure 4d and Figure 5a–c).

Another remarkable feature is the presence of globular structures associated to lignin condensation that were observed on the surface of samples treated with 90:10 and 50:50 ratios of EtOH/water (Figure 5a,b). When ethanol concentration increases in the liquid mixture, then clefts and defects appear on the surface of cell wall, which seems to be corroded. When sodium hydroxide is used as catalyst, the most evident effect on the bark surface is the separation of the cell bundles (Figure 4e), followed by total collapse and breaking up of the structure, resulting in a highly heterogeneous solid fraction.

## 3. Discussion

The compositional analysis of birch outer bark revealed a material mainly composed of the polyester suberin (44% *w*/*w*) and extractives (40% *w*/*w*), with a low content of polysaccharides (4.5% *w*/*w*) and lignin (9% *w*/*w*). These values are similar to those reported in the literature for birch outer bark [2]. Ash content (0.67%) was also similar to that reported previously [2,6]. Different pretreatment methods were applied in order to evaluate the potential fractionation of this biomass material. The higher obtained yield of dissolved substances from uncatalyzed treatments reached 27% of initial dry mass and increased only upon the addition of alkaline catalyst in the liquid mixture. When compared to solubilization rates of organosolv pretreated, under similar conditions, cork from *Quercus suber* L. [18], the yields appear significantly lower. This can be attributed to the rigid structure of bark or the higher extractives content of the bark feedstock (40% *w*/*w*) when compared to those from cork (19% *w*/*w*). Another possible reason could be that the presence of suberin in the cell walls can limit the diffusion of the liquid mixture [18] inside the bark structure. The recalcitrant ester bonds in suberin can be readily hydrolyzed by bases, as clearly shown by the substantial yield increase when sodium hydroxide was added to the liquid phase. Throughout the literature, various strategies have been developed for the pretreatment of bark biomass from different tree species both hardwood and softwood either as a part of the bioethanol production process or as a prehydrolysis method for the isolation of valuable compounds. Dilute sulfuric acid and alkaline pretreatment methods have been applied to pine and poplar bark in order to facilitate the enzymatic digestibility of cellulose to ethanol or to increase the phenolic content of spruce bark by removal of hemicelluloses [32,33,34]. Hydrothermal pretreatment has been proven an efficient method for the removal of hemicellulose from beech bark [35] and the increase of sugars yields from enzymatic hydrolysis of cellulose in *Eucalyptus* bark [36]. Finally, steam explosion has been utilized as a pretreatment method for the production of bioethanol from spruce bark, as well as for the extraction of the triterpene betulin from birch bark in combination with alkaline hydrolysis [37,38].

The Klason lignin content of the residual solid fractions increased after pretreatment, although a decomposition and removal of lignin aromatic structures can be assumed from FTIR and NMR data. Previous studies report that water-soluble phenolic compounds that are present in bark can condense with lignin during pretreatment and appear as acid-insoluble lignin in the subsequent compositional analysis [33,37,39]. Degradation products generated during the pretreatments as a function of the severity of the process and the concentration of carbohydrates present in outer bark were in low concentrations (0.2%–1.8% *w*/*w* of the initial bark dry matter). Release of acetic acid was more extensive when steam explosion and organosolv pretreatment are applied, following the release of hemicellulosic sugars detected in the liquid fraction.The ethanol/water ratio was shown to affect significantly disruption of lignin/hemicellulose ester bonds.

Since ATR-FTIR spectra can provide useful information for identifying the presence of certain functional groups or chemical bonds in a molecule or an interaction system, it was applied here to survey the changes in chemical structures before and after extraction by various methods. Bands at 2935–2850, 1740, 1265, 1161, 723 cm^−1^ were used for monitoring ester bonds and thus suberin presence and structure, while bands at 1513, 1468, 855 and 819 cm^−1^ were used for lignin aromatic vibrations. Apart from the samples after hydrothermal and treatment with alkali catalyst, there are no detectable changes in the ATR-FTIR spectra indicating suberin removal, while lignin removal is profound in all OS samples. The clear presence of signals from ester groups at 1740 cm^−1^, associated with the low intensity of signals from acidic groups at 1715 cm^−1^, indicates the polymeric nature of residual suberin in most of the samples. The latter band is associated with hydrogen bonded carbonyl groups in either esters or acids and reflects the formation of monomers released during the polymerization of suberin molecule. The presence of suberin can be also reflected by the strong intensity of the 1161 cm^−1^ band from C–O–C asymmetric stretching of high esters. However, one important observation is the reduced intensity of the 1265 cm^−1^ band from C–O–C symmetric stretching in all pretreated samples, compared to the 1161 cm^−1^ band, corroborating the idea that cleavage of ester bonds within the polymeric structure of suberin (ester bonds in epoxide groups, head-to-tail bonds between hydroxyl fatty acids and diacids) occured [30], while the resistant domain close to glycerol anchor parts and the phenolic components remained unaltered. Cleavage of bonds in the polysaccharidic part of the outer bark cell walls was more intense than for the aromatic and the ester part of the polymeric structure when SE and OS treatment was applied; however, the increase in band 1032 cm^−1^ relative to that at 1161 cm^−1^ suggests that lignin has been more largely removed in comparison to polysaccharides [18].

The NMR spectra of OS/NaOH residual solid show that both main aliphatic bands generally decrease, but the decrease at 33 ppm is more significant than that at 30 ppm, which confirms the preferential removal of these methylenes compared to those at 30 ppm. This is attributed to the presence of alkaline catalyst that cleaves the ester bonds closer to linkage groups, thus removing shorter aliphatic chains, while it may also account for the removal of a higher number of –CH_2_ groups close to the glycerol anchorage points compared to the middle- and end-chain –CH_2_. Upon 80:20 OS treatment, the opposite effect is observed; 30 ppm carbons resonances from polymethylenic domains are decreased, suggesting that higher concentration of ethanol in the pretreatment liquor leads to middle-and end-chain bonds cleavage. The reduction of 30 ppm carbons in NMR spectra of OS/EtOH samples goes along with the increase of ethanol concentration in the liquid mixture. ^13^C ssNMR molecular dynamics studies in other suberinic materials, such as cork and potato periderm, have also revealed the presence of two different populations of aliphatic CH_2_ (methylene) groups, complementing the idea that suberin rigid moieties correspond to the hydrocarbon chains in the more orderly organized parts of the suberin polyester, close to the the glycerol anchorage points, as well as those with more freedom of motion to the hydrocarbon chains in less constrained regions [30,31,40]. The marked loss of carbonyl signal at 172 ppm in all organosolv pretreated samples confirmed the cleavage of acetyl groups from suberin and hemicelluloses. In all spectra of samples treated with liquid mixture containing ethanol, a reduction of resonances attributed to lignin is detected. A clear presence of signal from the ester groups, along with the absence of resonances from aromatic groups, suggests that organic solvents penetrate the cell wall and remove lignin, with the effect on recalcitrant suberin bonds close to linkage groups being less intense.

Scanning electron microscopy was used in order to appreciate the morphological changes after pretreatment. The destruction of the cell layers and phellem configuration in the sample treated with alkaline catalyst is probably related to lignin removal. It is speculated that lignin is mainly located in the middle lamellae, the membrane delimiting neighboring cells; studies has shown that in wood cells at least 50% is concentrated in this compartment [41,42]. As a result, partial depolymerization of lignin may lead to separation of cell bundles. Collapse of cell wall can also occur after cleavage of lignin/hemicellulose ester bonds and subsequent suberin removal, which also occurs when alkaline treatment is applied. Higher amount of lignin droplets on the cell wall surface were observed for bark samples treated with 50:50 EtOH/water ratio compared to 90:10, probably due to the fact that higher concentration of organic solvent may draw away the lignin components formed during pretreatment in the liquid mixture, which can adhere to each other and deposit again on the biomass surface upon cooling [43]. The formation of lignin agglomerates on the surface has also been described in other lignocellulosic biomasses exposed to steam-explosion, diluted acid or organosolv pretreatments [44,45,46].

## 4. Materials and Methods

### 4.1. Sample

**Figure 6:**
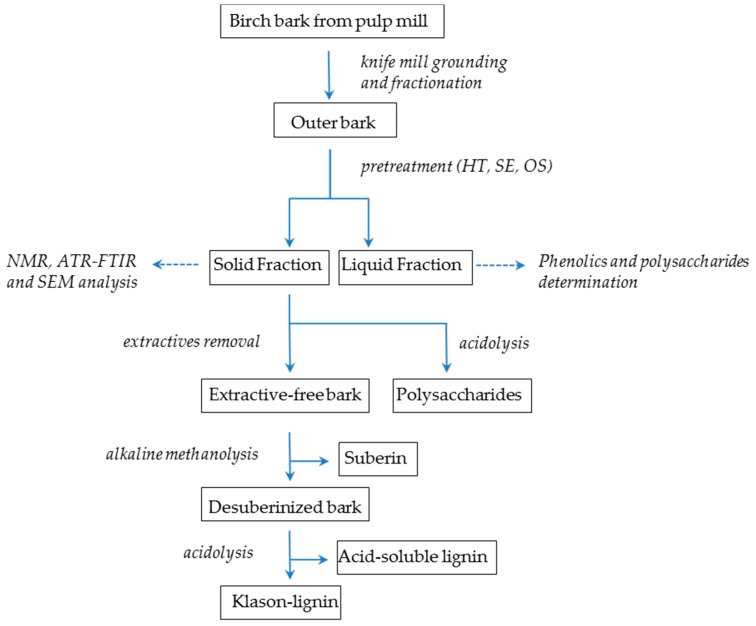
Flow diagram of the experimental steps followed.

The analytical protocol applied in this work is illustrated in Figure 6. The bark from *Betula pendula* was obtained as residual by-product of commercial debarking at Smurfit Kappa pulp mills (Piteå, Sweden) with 4.5% of moisture and was ground in a knife mill (Retsch SM 3000, Haan, Germany) using an output sieve of 1 mm × 1 mm. After grounding, dry birch bark samples were soaked by mixing in distilled water. The outer bark fraction, which floated to the top of the water surface, was collected and dried [6,47]. Final moisture was 2%–3%. The mass retained was weighed and stored in plastic bags until pretreatment. Ash content before and after pretreatment was determined with incineration of the material at 550 °C overnight and weighing of the residues.

### 4.2. Pretreatment

A summary of the processing conditions of each pretreatment is given in Table 5. Steam explosion pretreatment was carried out in a 12 L pressurized vessel of a pilot scale steam explosion reactor, installed in the Biochemical Process Engineering Laboratory, LTU, Luleå, Sweden. The liquid mixture consisted of water (SE sample) or water–EtOH in ratio 90:10 (OS/SE sample). The vessel was heated with steam to the desired temperature (195 °C/15 bar or 203 °C/21 bar when EtOH 10% was used) and maintained during the specific reaction time there before releasing the pressure to a collection vessel. Hydrothermal (HT) and organosolv (OS) pretreatments took place in a high-pressure hydrothermal reactor and have been conducted in replicates. A suspension of biomass-water (HT) or biomass-water-EtOH (OS), with a solid to liquid ratio 1:9, was heated to the reaction, kept at its set-point during the specific reaction time, and subsequently cooled down below 40 °C. In case of alkaline catalyst, sodium hydroxide was added to the liquid mixture to a final concentration of 0.1 M. After pretreatments, the pH of the resulting slurry was determined and the solid residue was washed until pH reached approximately 5.0 with warm distilled water before any further analysis. After filtration, the samples were dried in a vacuum oven and weighed to determine the solid recovery and calculate the % solubilization of bark.

The treatments were evaluated with the severity correlation, which describes the severity of the pretreatment as a function of treatment time (min) and temperature (°C): Log(R_o_) = Log (t exp(T − T_ref_)/14.7, where T_ref_ = 100 °C [48,49]. The effect of pH was taken into consideration by Combined Severity [50], where the pH of the liquor was employed as a measure of the hydrogen ion concentration for water and ethanol-water solutions: Combined Severity (CS) = Log(R_o_) − pH.

### 4.3. Chemical Characterization

Extractives were fully removed by successive Soxhlet extractions with dichloromethane, ethanol and water. The solvents were recovered and the extractives content determined from the mass of the solid residue after drying at 105 °C, and reported as a percentage of the original samples.

The extractive-free bark sample was used for determination of suberin by use of methanolysis for depolymerisation [51]. A 1.5 g sample of extractive-free material was refluxed with 100 mL of a 3% methanolic solution of NaOCH_3_ in CH_3_OH during 3 h. The sample was filtrated and washed with methanol; the residue was refluxed with 100 mL CH_3_OH for 15 min and filtrated again. The combined filtrates were acidified to pH 6 with 2 M H_2_SO_4_ and evaporated to dryness. The residues were suspended in 50 mL water and the alcoholysis products recovered with dichloromethane in three successive 50 mL dichloromethane extractions. The combined extracts were dried over anhydrous Na_2_SO_4_, and the solvent was evaporated to dryness. The suberin extracts, that include the fatty acid and fatty alcohol monomers of suberin, were quantified gravimetrically, and the results expressed in percent of the initial dry mass.

Klason and acid-soluble lignin, and carbohydrates contents were determined on the extracted and desuberinized materials after acidolysis. Sulphuric acid (72%, 3.0 mL) was added to 0.3 g of the material sample, and the mixture was placed in a water bath at 30 °C for1 h after which the sample was diluted to a concentration of 3% H_2_SO_4_ and hydrolyzed for 1 h at 120 °C. The sample was vacuum-filtered through a crucible, washed with boiling purified water and Klason lignin was determined as the mass of the solid residue after drying at 105 °C. The acid-soluble lignin was determined on the combined filtrate following TAPPI T 222 and TAPPI UM 250 methods by measuring the absorbance at 205 nm using a UV/Vis spectrophotometer (SpectraMax 250 Microplate reader, Molecular Devices, Sunnyval, CA, USA) and using an extinction coefficient of 110 L·g^−1^·cm^−1^.

The polysaccharides were determined after acidolysis of the extractive-free material, as described above. The combined filtrates were further subjected to complete digestion by incubating 4.5 mL of each sample with 0.5 mL HCl 4 M at 70 °C for 15 min followed by neutralization with 0.5 mL NaOH·12N. The monomeric sugars produced were quantified with isocratic ion-exchange chromatography using an Aminex HPX-87P column with a De-Ashing Bio-Rad micro-guard column at 85 °C (Bio-Rad Laboratories, Hercules, CA, USA) using Millipore water at a flow rate of 0.5 mL·min^−1^ as the mobile phase. Organic acids and other by-products in pretreated materials were determined using an Aminex HPX-87H chromatography column with a Cation-H Bio-Rad micro-guard column at 65 °C (Bio-Rad Laboratories), with a mobile phase of 5 mM sulphuric acid at a flow rate of 0.6 mL·min^−1^.

The total phenolic content in the hydrolysate (liquid phase) of the pretreated materials was determined using Folin–Ciocalteu method. Gallic acid (GA) was used as standard and the total phenolic content was expressed as mg GA equivalent/mL. An aliquot (50 μL) of each extract, or of the gallic acid standard, was mixed with 2 mL of Folin–Ciocalteu reagent (1:10 *v*/*v*). After 3 min, at room temperature, 2 mL of aqueous Na_2_CO_3_ (7.5% *m*/*v*)was added, then the mixture was vortexed and further incubated in a thermostat at 45 °C for 15 min and the absorbance of the resulting blue colored mixtures was recorded at 765 nm against a blank containing only water.

### 4.4. ATR-FTIR Measurements

ATR-FTIR spectra of extractive—free untreated and pretreated solid fractions were collected on a Bruker IFS 66v/S FTIR spectrometer (Bruker Daltonics, Billerica, MA, USA) using a single reflection ATR cell (DuraDisk, equipped with a diamond crystal). Data were recorded at room temperature, in the range of 4000–400 cm^−1^, by accumulating 128 scans with a resolution of 4 cm^−1^. Three replica spectra were collected for each sample in order to evaluate reproducibility (OPUS v6.0; Bruker Daltonics, Billerica, MA, USA).

### 4.5. ^13^C/MAS NMR Measurements

^13^C-NMR analyses were recorded with a Bruker Ascend Aeon WB 400 spectrometer. Data extractive—free untreated and pretreated solid fractions were obtained at 25 °C using standard Bruker pulse programs. ^13^C-CP/MAS NMR spectra were recorded at 9.48 T using 12 kHz spinning rate and MAS with proton 90° pulses 2.5 µs. Chemical shifts are given in ppm from glycine. The NMR spectra were processed and analyzed with Topspin software (3.5; Bruker BioSpin Scandinavia, Solna, Sweden).

### 4.6. Scanning Electron Microscopy (SEM)

Samples from extractive—free untreated and pretreated materials were dried prior to use and coated with a thin layer of tungsten using a sputter coater. Electron micrographs were recorded using the extreme high-resolution (XHR) FEI Magellan™ 400 system (FEI Company, Eindhoven, The Netherlands) operated at 3.0 kV. Micrographs are regarded as representative.

## 5. Conclusions

The present work studies the chemical composition of the European hardwood *Betula pendula* silver birch barks after different pretreatment methods. The solid fractions obtained present different characteristics depending on the specific process conditions. During hydrothermal and steam explosion pretreatment, cleavage of the hemicellulosic bonds led to the release of hemicellulosic sugars in the liquid fraction, while suberin was partially depolymerized. Organosolv treatment with ethanol/water solvent was proved to be an efficient method for the fractionation of bark components, resulting in removal of significant extractives, cleavage of lignin/hemicellulose bonds, removal of lignin and retaining of suberin components. This work contributes to a deeper fundamental knowledge of the bark chemical composition and the development of pretreatment strategies for a selective component enrichment to facilitate downstream fractionation processes and bark valorization.

## Figures and Tables

**Table 1:** Severity factor (SF)/combined severity (CS) and % dissolved bark for different pretreatment methods.

Batch	Type	SF/CS	Bark Solubilization %
#1	HT	3.94	12.00
#2	SE	3.97	23.33
#3	OS/SE	4.81	21.67
#4	OS 50:50	4.81	21.00
#5	OS 80:20	4.15	26.88
#6	OS/NaOH	4.15	61.25

**Table 2:** Summative chemical composition (% of total dry weight) of untreated and residual solid fraction of pretreated bark samples.

	Untreated	HT	SE	OS/SE	OS 50:50	OS 80:20	OS/NaOH
**total sugars**	**4.45**	**5.38**	**5.61**	**6.11**	**6.19**	**7.58**	**7.75**
glucose	2.19	4.35	4.43	5.37	4.26	4.86	5.82
xylose	0.67	0.58	0.80	0.53	1.28	0.75	0.61
galactose	0.21	0.06	0.03	n.d.	n.d.	n.d.	n.d.
arabinose	1.21	0.35	0.28	0.10	0.63	0.11	0.18
mannose	0.18	0.04	0.07	0.11	0.02	n.d.	0.01
**ash**	**0.67**	**0.41**	**0.26**	**0.19**	**0.19**	**0.23**	**0.21**
**total extractives**	**39.39**	**27.98**	**31.58**	**30.19**	**29.23**	**22.32**	**19.27**
dichloromethane	25.49	24.57	26.99	25.65	23.76	16.18	16.56
ethanol	9.96	2.67	1.82	2.69	1.18	3.89	1.21
water	3.94	0.74	2.77	1.85	4.29	2.25	0.5
**suberin**	**44.06**	**40.47**	**38.33**	**35.16**	**42.01**	**34.52**	**29.94**
**lignin**	**9.11**	**20.97**	**19.84**	**20.93**	**16.36**	**25.57**	**26.5**
klason	8.87	20.80	19.59	20.65	16.18	25.36	26.2
acid-soluble	0.24	0.17	0.25	0.27	0.18	0.21	0.3
**suberin + lignin**	**53.17**	**61.44**	**58.17**	**56.09**	**58.37**	**60.09**	**56.44**
**total**	**97.68**	**95.21**	**95.62**	**92.58**	**93.98**	**90.22**	**83.67**

n.d. Not detected.

**Table 3:** Composition of the liquid fraction (g/L of liquid fraction) of the pretreated bark samples.

	HT	SE	OS/SE	OS 50:50	OS 80:20	OS/NaOH
**total sugars**	**0.39**	**2.02**	**2.41**	**1.14**	**1.69**	**2.16**
glucose	0.21	0.17	0.21	0.53	0.42	0.31
xylose	0.09	0.33	0.43	0.13	0.31	0.45
galactose	0.04	0.17	0.38	0.03	0.13	0.12
arabinose	0.02	1.04	0.69	0.45	0.85	1.28
mannose	0.01	0.09	n.d.	n.d.	n.d.	n.d.
**phenolic compounds**	**0.13**	**0.20**	**0.27**	**0.74**	**2.74**	**5.74**
**inhibitors**						
formic acid	n.d.	0.25	0.90	0.16	0.67	0.67
acetic acid	2.16	1.13	2.97	0.67	1.69	2.11
levulininc acid	n.d.	n.d.	n.d.	n.d.	n.d.	n.d.
HMF ^α^	0.37	0.05	0.46	0.07	0.06	0.12
furfural	0.61	0.18	1.13	0.06	0.09	0.27

^α^ 5-Hydroxy-methyl-furfural; n.d. Not detected.

**Table 4:** ^13^C-NMR spectra assignments of the functional groups identified in suberinic material.

^13^C δ/ppm	Functional Group	Assignment [22,24,28,29,30,31]
29–30	CH_2_	aliphatic methylenic groups
32–33	CH_2_COO; CH_2_COOH	methylene linked to carboxylic moieties
56	CH; OCH_3_	epoxy ring; methoxy groups
64	CH_2_OH;CHOH	Alcohols
72	OCH	methyne adjacent to ester groups
74	OCH_2_	methylene adjacent to ester groups
130	CH=CH	vinylic groups
144–145	-C=	aromatic groups
172	COO; COOH	ester and carboxylic acid groups

**Table 5:** Processing conditions of different types of pretreatment applied to birch outer bark.

Batch	Type	Catalyst	Catalyst Concentration (M)	Ethanol/Water	Temperature (°C)	Time (min)
#1	HT	-	-	-	200	10
#2	SE	-	-	-	195	10
#3	OS/SE	-	-	10/90	203	60
#4	OS 50:50	-	-	50/50	195	60
#5	OS 80:20	-	-	80/20	160	240
#6	OS/NaOH	NaOH	0.1	80/20	160	240

## Abbreviations

The following abbreviations are used in this manuscript:

HT	Hydrothermal pretreatment
SE	Steam Explosion
OS	Organosolv pretreatment
ATR-FTIR	Attenuated Total Reflectance—Fourier Transform Infrared Spectroscopy
NMR	Nuclear magnetic resonance
SEM	Scanning Electron Microscopy
